# The Stark law, from inception to COVID-19 blanket waivers: a review

**DOI:** 10.1186/s13037-022-00326-9

**Published:** 2022-06-02

**Authors:** Amrita Shenoy, Gopinath N. Shenoy, Gayatri G. Shenoy

**Affiliations:** 1 Healthcare Administration, University of Baltimore, College of Public Affairs, School of Health and Human Services, Healthcare Administration Program, 1420 N. Charles Street, Baltimore, MD 21201 USA; 2 University of Mumbai, K J Somaiya Medical College and Hospital, College of Physicians and Surgeons of Bombay, Consumer Disputes Redressal Forum, Mumbai Suburban District, State Government of Maharashtra, Mumbai, India; 3 National Board (DNB) Faculty of Anesthesiology, K J Somaiya Medical College and Hospital, Mumbai, Maharashtra India

**Keywords:** Stark Law, Stark I, Stark II, Designated Health Services, Financial Relationship, Remuneration from Self-referrals, Safe Harbors, Sanctions, Temporary COVID-19 Stark Law blanket waivers

## Abstract

The concept of physicians referring patients to their own healthcare entities is considered a “self-referral”. A discerning factor of a self-referral is when the physician has a financial interest in the entity of patient referral. Prospects of healthcare overutilization and costs, thereby, rise. Self-referral laws, therefore, are important to regulate overutilization and contain costs. In the 1980s, Congressman Fortney Stark initiated an act that was one of the precursors to one such self-referral law, known as the Stark Law. The Stark Law, in its initial phase, known as Stark I, addressed self-referrals selectively from laboratory services. Stark I, thereafter, in a series of subsequent amendments and enactments, burgeoned to include multiple services, referred as Designated Health Services (DHS), for self-referrals. The expanded law, inclusive of those DHS, is now known as Stark II. The passage of the 2010 Affordable Care Act as well as the prevailing 2019 Coronavirus Disease (COVID-19) pandemic further modified the Stark Law. Given the legislative history of the said law, the present review curates the legal initiatives of this law from its nascent formative stages to the present form. The purpose of the above curation is to present a bird’s eye view of its evolution and present analysts of any future research segments. This review, furthermore, describes the waivers of this law specific to COVID-19, or COVID-19 blanket waivers, which are instruments to assuage any barriers and further placate any hurdles arising from this law prevalent in this pandemic.

## Background

Caring for the sick and ailing often involves the expertise of more than one physician. In the continuum of care, physicians refer their patients to other clinical specialists. The practice of referring patients is broadly described as a patient referral. Physicians are considered to make referrals, directly or indirectly, if they caused, directed, or controlled referrals made by others [1].

A “self-referral” refers to the practice of physicians referring their patients for medical treatment or services to an entity in which either the physician or an immediate family member of the physician has a financial interest [2]. In 1972, self-referrals, initially, drew attention with the Medicare Fraud and Abuse statute [3]. Ever since, amendments, legislations, and regulations to patient self-referral laws prevail and continue to develop, as with the more recent Coronavirus Disease-19 (COVID-19) pandemic of today.

In 1989, Democratic Congressman, Fortney (Pete) Stark, then chairman of the House Ways and Means Subcommittee on Health, stated, “the integrity of our nation’s physicians is being threatened by seductive deals promoted by fast buck artists. Further proliferation of these ventures is bound to undercut public confidence in the medical profession.” [3, 4]. In 1989, Congressman Stark, then, reintroduced the Ethics in Patient Referrals Act [3–6]. The intent of the above act was to contain and regulate self-referrals to reduce treatment costs for patients.

Self-referral laws were, thereby, enacted to protect the patient’s best interest. The above laws safeguard that the patient receives the most efficacious treatment at an affordable cost. There were many reasons to enact and implement physician self-referral laws. Enacting and implementing physician self-referral laws is a necessary step in securing its regulation. The above laws, moreover, are perceived as instruments in curbing the overutilization of healthcare services.

The presence of self-referral laws, concurrently, appear to promote physicians’ better gauging the medical necessity of a referral or service [3, 5]. The patient, thereby, benefits from eliminating expensive and unnecessary services at entities in which they are treated [3]. Of the reasons known for regulating self-referrals, a particular law contextual to physician self-referrals is the focus of this paper. The above law that selectively pertains to physician self-referrals is known as the Physician Self-Referral Law (or hereinafter, the Stark Law).

The Centers for Medicare & Medicaid Services (CMS) specify the Stark Law stated in Sect. 1877 of the Social Security Act [7]. The said law: (1) prohibits a physician from making a referral for certain designated health services (DHS) payable by Medicare to an entity with which he or she (or an immediate family member) has a financial relationship, unless an exception applies and its requirements are satisfied [7], (2) prohibits the entity from filing claims with Medicare (or billing another individual, entity, or third-party payer) for those referred DHS, and (3) establishes a number of specific exceptions and grants the Secretary the authority to create regulatory exceptions for financial relationships that do not pose a risk of program or patient abuse [7].

## Core elements of the Stark Law

The Stark Law has a set of well-defined seven core elements [8]. Those core elements are stated as follows: (1) physician, (2) referral, (3) designated health service(s) (DHS), (4) entity, (5) financial relationship, (6) exceptions, and (7) statutory penalties [8]. A physician means a doctor of medicine or osteopathy, dentist, podiatrist, optometrist, or chiropractor [8]. Providers such as nurse practitioners, physician’s assistants, and physical therapists are not included within this definition [8].

A “referral” is the request by a physician for, the ordering of, or the certifying or recertifying of the need for, any DHS, including the request for a consultation with another physician and any test or procedure ordered by or to be performed by (or under the supervision of) that other physician [9, 10]. A “referral” does not include DHS personally performed or provided by the referring physician [9, 10]. Table 1 tabulates the list of DHS currently applicable under the above law [7, 11].

**Table 1:** List of Designated Health Services

1	Clinical laboratory services,
2	Physical therapy services,
3	Occupational therapy services,
4	Outpatient speech-language pathology services,
5	Radiology and certain other imaging services,
6	Radiation therapy services and supplies,
7	Durable medical equipment and supplies,
8	Parenteral and enteral nutrients, equipment, and supplies,
9	Prosthetics, orthotics, and prosthetic devices and supplies,
10	Home health services,
11	Outpatient prescription drugs, and
12	Inpatient and outpatient hospital services

Source: https://www.cms.gov/Medicare/Fraud-and-Abuse/PhysicianSelfReferral/List_of_Codes

An entity is referred to any person or entity that performed DHS that are billed as DHS and those who billed DHS to Medicare or Medicaid for reimbursing a bill or claim [8]. A financial relationship is construed as one of the following four [8]: (1) an ownership interest, (2) an investment interest, or (3) a compensation arrangement between the physician (or a physician’s immediate family member) and the entity, and (4) a compensation arrangement that may be either direct or indirect [8].

On the one hand, a direct financial relationship exists if remuneration passes between the referring physician (or a member of his or her immediate family) and the DHS furnishing entity without any intervening persons or entities in-between the above DHS entity and the referring physician (or a member of his or her immediate family) [8].

On the other hand, an indirect ownership or investment interest exists when there is an unbroken chain of owners between the referring physician and the DHS entity, and the latter has actual knowledge of (or acts in reckless disregard or deliberate ignorance of) the fact that that the referring physician has some ownership or investment interest in the above entity [8].

The Stark Law has certain exceptions for financial relationships and/or arrangements [8]. The above law is not invoked when certain transactions fall within the purview of these exceptions. Table 2 tabulates the list of Stark Law exceptions [12].

**Table 2:** List of Financial Exceptions under the Stark Law

1	Owning stocks or bonds in a large, publicly traded company or mutual fund,
2	Owning or investing in certain rural providers or hospitals in Puerto Rico,
3	Reasonable rent for office space or equipment,
4	Amounts paid under fair and bona fide employment relationships,
5	Reasonable payments for personal services provided to the entity or for other services unrelated to the provision of designated health services,
6	Compensation under a legitimate “physician incentive plan,” such as by withholds, capitation, or bonuses in managed care,
7	Reasonable payments to induce a physician to relocate to the hospital’s service area,
8	Isolated transactions, such as a one-time sale of property or a practice,
9	An arrangement that began before December 19, 1989, in which services are provided by a physician group but are billed by the hospital, and
10	Reasonable payments by a physician for clinical laboratory services or for other items or services

Source: Showalter JS. The Law of Healthcare Administration. Health Administration Press; 2012

Claims for DHS submitted in violation of the Stark Law triggers the following sanctions: denial of payment requiring amounts received to be refunded [8, 10], civil monetary penalties of $15,000 per service in which the violation is known [8, 10], and exclusion from Medicare and Medicaid programs in which a physician or entity has knowingly entered into an improper cross-referral arrangement or scheme designed to circumvent the self-referral prohibition [8, 10], and monetary penalties of up to three times the amount of the claim plus a penalty of an amount between $5,000 and $10,000 per claim [8, 10].

A review of past literature presented the initiation, legislative developments, and regulatory history, and thereby, cumulated the evolution of the Stark Law from 1972 to 2006 [3]. Thereafter, numerous amendments of the above law have taken place owing to the rapidly evolving healthcare landscape. As examples, both the 2010 Affordable Care Act (ACA) and the ongoing COVID-19 pandemic has initiated and made effective specific changes of the above law.

This paper has three research questions giving rise to three objectives. First, this review paper sequentially curates the above law’s evolution, from initiation to 2020. This is an attempt to chronologically tabulate its updates. Second, the present review describes initiatives and functions pertaining to the evolution of the above law. Finally, it describes how the 2020 COVID-19 Stark Law blanket waivers (hereinafter, blanket waivers) support specific patient self-referrals, expand on the permissible ways of remuneration for self-referrals, and protect eligible physicians from legal sanctions contextual to the COVID-19 pandemic.

There are two-fold objectives to curating developments of the Stark Law. First, to update past literature. Second, to further assist policy analysts identify any areas within the above law that may need amendments, if need be, as per their discretion, in the future. Finally, to discern how these blanket waivers financially and medicolegally buffer eligible physicians during the ongoing national and public health emergencies.

## Stark Law blanket waivers during the COVID-19 pandemic

Section 1135 of the Social Security Act authorizes the Secretary of the Department of Health and Human Services to waive or modify certain requirements of healthcare programs such as Medicare, Medicaid, Children’s Health Insurance Program (CHIP), and Health Insurance Portability and Accountability Act (HIPAA) of 1996 [13].

There are two prerequisites that need to be met before the Secretary invokes the authority to waive or modify certain requirements of the above programs [13]. Those two prerequisites are: (1) the President must have declared an emergency or disaster under either the Stafford Act or the National Emergencies Act [13], and (2) the Secretary must have declared a Public Health Emergency under Sect. 319 of the Public Health Service Act [13].

On March 13, 2020, President Donald J. Trump proclaimed a nationwide emergency pursuant to Sec. 501(b) of the Stafford Act [14]. On January 31, 2020, prior to the above Presidential declaration, Alex M. Azar II, Secretary of the Department of Health and Human Services (DHHS), determined under Sect. 319 of the Public Health Service (PHS) Act, that a nationwide public health emergency exists and has existed since January 27, 2020 [15].

On March 13, 2020, therefore, with both the Presidential proclamation and DHHS Secretary’s determination of the nationwide emergency and public health emergency, respectively, both the above prerequisites were met to invoke the authority to waive or modify certain requirements of the above healthcare programs [13].

On March 30, 2020, consequently, DHHS Secretary used his authority under the Sect. 1135 of the Social Security Act to waive certain requirements of the healthcare programs [13]. These waivers, more specifically, comprise of those for Sect. 1877(g) of the Social Security Act (or the Stark Law) [13]. CMS, on the very same day, thereby, issued waivers under the Stark Law, given the national and public health emergencies [16].

On March 30, 2020, CMS, therefore, issued the set of 18 blanket waivers to the Stark Law [16]. These waivers are retroactively effective on March 1, 2020, related purely to COVID-19 purposes, and would terminate after a certain duration as per Sect. 1135(e) [13]. Section 1135 waivers expire at the end of the national emergency period or 60 days from when they are effective [17]. These blanket waivers, therefore, need to be renewed if a need persists to extend its applications [17].

## Discussion

Table 3 presents a bird’s eye view of the legislative and regulatory evolution of the Stark Law from 1972 to 2020 [3, 13, 18–27]. Table 4 presents 18 blanket waivers that CMS issued during the COVID-19 pandemic [13].

**Table 3:** Curated developments of the Stark Law

#	Year	From Stark I to Stark II: Evolution over Time		
		**Developments**	**Details**	**Function(s)**
1	1972	Medicare Fraud and Abuse Statutes were introduced [3]	Regulations regarding fraud and abuse began with this Federal statute. It further contained anti-kickback provisions [3]	To contain knowing and willful compensation from self- referrals
2	1977	Medicare Fraud and Abuse Statutes expand [3]	This expansion includes safe harbors which are certain types of permissible remunerations [3]	To expand the antikickback laws and upgrading violations from misdemeanor to felony offenses [3]
3	1982	Federal Regulation prohibiting referrals to a Medicare certified Home Health Agency was enacted [3]	This targeted financial interest arising from Medicare Home Health Agency Referrals [3]	To prevent physicians from referring patients to a Medicare certified home health agency if there was a direct or indirect financial interest [3]
4	1987	The Medicare and Medicaid Patient & Program Protection Act was introduced [3]	This targeted financial interest arising from false claims, forbidden business transactions, excessive charges, and remuneration from referrals [3]	To contain prohibitions against false claims for reimbursement, failures to report forbidden business transactions, excessive charges, and remuneration for referrals [3]
5	1988	The Ethics in Patient Referrals Act (H.R. 5198) was first introduced [3]	Democratic Congressman Fortney Pete Stark initiated and introduced this Act [3]	To prohibit physician self-referrals, reduce costs thus incurred by these arrangements to Medicare and its beneficiaries [3]
6	1989	The Ethics in Patient Referrals Act (H.R. 939) was reintroduced [3]	Congressman Stark reintroduced HR 939 [3]	This Act prohibited physicians from referring Medicare patients to healthcare entities in which they have ownership or financial interest [3]
7	1989	The Ethics in Patient Referrals act was passed (December 1989) [3]	The Ethics in Patient Referrals Act passed as part of HR 3299-The Omnibus Budget and Reconciliation Act of 1989 (OBRA 89). Stark I was included in Sect. 6204 of OBRA 89 as Public law 101–239. Section 6204 of OBRA 89, thereafter, added Stark I as Sect. 1877 to the Social Security Act (SSA) [3]	To prohibit physicians from referring Medicare beneficiaries to clinical laboratories in which the former or their immediate family members have financial interests in those laboratories [3]
8	1991	The Subcommittee on Health, the Subcommittee on Oversight and the Subcommittee on Ways and Means heard testimony from researchers on the status of physician ownership of healthcare facilities other than clinical laboratories [3]	This testimony served as a foundation for expanding physician self-referral laws	There was a discussion of physician ownership in healthcare entities other than that of clinical laboratories [3]
9	1992	Stark I becomes effective (January 1992) [3]	Stark I was expanded in 1993 after it became effective [3]	To include the new Stark II provisions as detailed in Sect. 13,562 of COBRA 93 [3]
10	1993	The Comprehensive Physician Ownership and Referral Act of 1993 (HR 345) [3]	Congressman Stark introduces HR 345. HR 345 did not pass [3]	To extend the Medicare ban on physician referrals to providers with whom the former has a financial relationship, include entities other than clinical laboratories, and modify safe harbors/Medicare provisions related to financial arrangements [3]
11	1993	Stark II is included in Sect. 13,562 of OBRA 93 (January 1993) [3]	The language of HR 345 was adopted in a diluted form in Sect. 13,562 of OBRA 93 that was known as Stark II [3]	To revise the provisions of Sect. 1877 of the SSA and include ten additional Designated Health Services (DHS). Self-referral laws were also made applicable to the Medicaid program [3]
12	1993	H.R 2264-OBRA 93 was introduced in the 103rd Congress (May 1993) [3]	Democratic Congressman Martin Olav Sabo introduced this bill. The above bill passed the House with a vote of 219 years to 213 nays. The Senate amended it in June 1993. At the Senate, the vote was equally divided. Upon Democratic Vice President Gore’s leaning yea vote this bill passed [3]	The proposed bill included Physician Ownership and Referral, which is currently referred to as Stark II [3]
13	1993	Stark II becomes Public Law # 103–66 (August 1993) [3]	President Clinton signed bill HR 2264-OBRA 93 to enforce as Stark II Law [3]	Stark II prohibits a physician with a prohibited financial relationship from referring a Medicare patient to an entity that provides a DHS. The entity is restricted from furnishing a Medicare reimbursement claim or bill to any individual, third part payer, or any other entity [3]
14	1994	The Social Security Amendments of 1994 were effective [3]	There were amendments to Social Security [3]	To amend the list of DHS, changed reporting requirements, and modified some of the effective dates [3]
15	1998	The CMS publishes a proposed rule to implement Stark II (January 1998) [3]	Public comments to the proposed rule led to a two- phased rule making process, Phase I and Phase II [3]	To apply the provisions of Stark II Law to applicable entities and physicians
16	2001	Stark II Phase I final rules and regulations were issued (January 2001) [3]	Phase I addressed the definitions applicable to the Stark Law, general prohibitions, in-office ancillary exceptions, the impact on physician group practices, and financial relationships between physicians and entities that provide DHS [3]	As the 1^st^ of the two phases to issue and implement Stark II
17	2002	Stark II Phase I final rules and regulations were effective (January 2002) [3]	Phase I regulations may be found in the Federal Register at 42 CFR, parts 411 and 424 [3]	The definitions applicable to the Stark Law, general prohibitions, in-office ancillary exceptions, the impact on physician group practices, and financial relationships between physicians and entities that provide DHS were effective Jan 2002 [3]
18	2004	CMS issued the Phase II interim final rules with a comment period (March 2004) [3]	Stark II Phase II regulations may be found in the Federal Register at 69 FR 16,054 [3]	To address statutory exceptions related to ownership and investment interests, compensation arrangement exceptions, and reporting requirements [3]
19	2004	Stark II regulations and Phase II rules were effective (July 2004) [3]	It also addressed public comments from Phase I and created new regulatory exceptions [3]	To implement Stark II Phase II [3]
20	2005	CMS published a regulatory text inclusive of C.ER.12 §§ 411.357(v) relating to exceptions for arrangements involving donations of electronic prescribing [18]	For ease of reference, CMS republished the entire Stark regulatory text as part of the Phase III final rule but omitted two exceptions of which this is the first [18]	This exception was published and finalized in 2005 [18]
21	2006	CMS published a regulatory text inclusive of C.ER.12 §§ 411.357(w) (2006) relating to exceptions for arrangements involving electronic health records technology [18]	For ease of reference, CMS republished the entire Stark regulatory text as a part of the Phase III final rule but omitted two exceptions of which this is the second [18]	This exception was published and finalized in 2006 [18]
22	2007	CMS issues the Phase III of the new final rule with a comment period (September 2007) [18]	Phase III addressed public comments from Phase II and thus, addressed the entire sent of comments from Phases I, II, and III [18]	To elaborate on previous discussions, not to change the scope or meaning [18]
23	2007	CMS proposed and issued several amendments to the Stark regulations in the 2008 Medicare Proposed Physician Fee Schedule (MPPFS) (July 2007) [19]	It identified certain issues for further studies and potential change in a separate rulemaking process throughout the preamble of the Phase III rulemaking [19]	These MPPFS proposals are separate from, and in addition to, the revisions in the Phase III final rule [19]
24	2008	CMS published final Stark rules (Final Rule) in its 2009 Final Hospital Inpatient Prospective Payment Systems Rule (August 2008) [19]	In the Final Rule, CMS makes various revisions to the Stark regulations. Some of these revisions emanate from proposals contained in the 2008 Medicare Proposed Physician Fee Schedule’ and some of the revisions emanate from proposals contained in the 2009 Inpatient Prospective Payment System Proposed Rule [19]	The Final Rule contains several significant modifications to the Stark regulations, some of which required physicians, hospitals, and other healthcare providers to unwind or restructure their arrangements [19]
25	2009	CMS almost adds an exception to the Stark Law as part of the 2009 Medicare Physician Fee Schedule [1]	This exception included incentive programs for both pay for performance and shared savings/gainsharing arrangements [1]	Adding this exception would have permitted hospitals to have incentive payment programs. However, CMS decided not to finalize this exception [1]
26	2010	The 2010 Affordable Care Act (ACA) makes many important changes to the Stark Law [20]	The ACA eliminates the Whole Hospital Exception, makes physicians subject to notice requirements when referring patients for MRI, CT or PET scans, and enforces a new self-reporting protocol for violations of the Stark Law. The above referral notice to patients must contain disclosures of the physician’s ownership interest, the patient’s option to receive services from other provider, and a list of alternative providers in the area [20]	To promote fair trade practices, curb over utilization, and provide patients an array of provider choices to receive for healthcare services [20]
27	2015	CMS releases final revisions to the Stark Law (the “Final Rule”) as part of the 2016 Physician Fee Schedule (November 2015) [21]	The Final Rule builds upon, and largely adopts, the similar July 8, 2015 proposed Stark rule (“Proposed Rule”) [21]	To accommodate healthcare delivery/payment system reform, reduce burdens, facilitate compliance, clarify certain applications of the Stark Law, and issue new Stark exceptions [21]
28	2016	CMS’s amendment to the Stark Law becomes effective (January 2016). A new exception called the Timeshare Arrangement is additionally added [22]	Timeshare Arrangements reduce the burden on healthcare providers and facilitate compliance with regulations, improve access to healthcare services, especially in underserved areas, and add flexibility to healthcare providers and ensure that it does not pose any risk to the patients [22]	To add much-needed flexibility for independent physicians who share office space and for hospitals that provide office space, equipment, personnel, supplies, and services to part-time, independent physicians on an “as-needed” basis [22]
29	2017	A bill titled Medicare Care Coordination Improvement Act of 2017 was introduced in both the House and Senate [23]	No further action was taken during this legislative session other than the referral to various committees [23]	To amend provisions (inclusive but not limited to) Title XVIII of the SSA to modernize the physician self-referral prohibitions, promote care coordination in the merit- based incentive payment system, and to facilitate physician-practice participation in alternative payment models under the Medicare program [23]
30	2018	CMS issued a Request for Information (RFI) seeking comments from the public on how to reform the Stark Law in response to the Trump Administration’s push to simplify administrative regulations that are impeding health care delivery (June 2018) [23]	CMS posed 20 requests for information regarding the Stark Law, asked for comments regarding concerns of the applicability of existing Stark exceptions, the ability to enter commercial alternative payment models, and the ability to enter novel financial relationships. CMS sought comment on whether any additional exceptions would be necessary to protect entities and individuals participating in these alternative payment models [23]	The RFI also sought feedback regarding the specific language in the current law, including “fair-market value,” “commercial reasonableness,” and “considers the volume or value of referrals.” CMS requested information as to the positive and negative effects of the Stark Law. The RFI was so far-ranging that CMS effectively invited comments on every aspect of Stark law that a stakeholder believed warranted revision or clarification [23]
31	2019	CMS issued a Modernizing and Clarifying the Physician Self-Referral Regulations Proposed Rule (Oct 2019). The deadline for comments to be considered was December 31, 2019 [24]	The proposed rule includes a comprehensive package of proposed reforms to modernize the regulations that interpret the Stark Law while continuing to protect the Medicare program and patients from bad actors. Under this proposed rule, for the first time, the regulations would support the necessary evolution of the American healthcare delivery and payment system [25]	The proposed rule supports the CMS “Patients over Paperwork” initiative by reducing unnecessary regulatory burden on physicians and other healthcare providers while reinforcing the Stark Law’s goal of protecting patients from unnecessary services and being steered to less convenient, lower quality, or more expensive services because of a physician’s financial self-interest [25]
32	2020	CMS issued blanket waivers of sanctions under the Stark Law, retroactive to March 1, 2020, in response to the COVID-19 pandemic [13]	CMS issued provider-specific guidance on how the Stark Law blanket waiver will impact physicians and other clinicians [26]	To protect only remuneration and referrals that are related COVID-19
33	2020	CMS published the final rule, “Modernizing and clarifying the Physician Self-Referral Regulations” in the Federal Register (December 2020) [27]	CMS finalizes many of proposed policies from the notice of proposed rulemaking issued in October 2019, including finalizing new, permanent exceptions for value-based arrangements, finalizing additional guidance on key requirements of the exceptions to the physician self-referral law, finalizing protection for non-abusive, beneficial arrangements, and reducing administrative burdens that drive up costs [27]	This rule had functions of (inclusive but not limited to) permitting physicians and other health care providers to design, entering into value-based arrangements without fear that legitimate activities, to coordinate and improve the quality of care for patients and lower costs would violate the Stark Law, to make it easier for physicians and other health care providers to make sure they comply with the law, and to safeguard the integrity of the health care ecosystem by taking money previously spent on administrative compliance and redirecting it to patient care [27]

**Table 4:** COVID-19 Blanket Waivers

Pursuant to Sect. 1135(b) of the Social Security Act, (42 U.S.C. § 1320b-5), I, the Secretary of Health and Human Services, hereby waives the sanctions and regulations under the Stark Law for referrals and claims related to the following, absent the government’s determination of fraud or abuse (Effective March 1, 2020):	
**#**	**Specifics of COVID-19 Blanket Waivers**
1	Remuneration from an entity to a physician (or an immediate family member of a physician) that is above or below the fair market value for services personally performed by the physician (or the immediate family member of the physician) to the entity
2	Rental charges paid by an entity to a physician (or an immediate family member of a physician) that are below fair market value for the entity’s lease of office space from the physician (or the immediate family member of the physician)
3	Rental charges paid by an entity to a physician (or an immediate family member of a physician) that are below fair market value for the entity’s lease of equipment from the physician (or the immediate family member of the physician)
4	Remuneration from an entity to a physician (or an immediate family member of a physician) that is below fair market value for items or services purchased by the entity from the physician (or the immediate family member of the physician)
5	Rental charges paid by a physician (or an immediate family member of a physician) to an entity that are below fair market value for the physician’s (or immediate family member’s) lease of office space from the entity
6	Rental charges paid by a physician (or an immediate family member of a physician) to an entity that are below fair market value for the physician’s (or immediate family member’s) lease of equipment from the entity
7	Remuneration from a physician (or an immediate family member of a physician) to an entity that is below fair market value for the use of the entity’s premises or for items or services purchased by the physician (or the immediate family member of the physician) from the entity
8	Remuneration from a hospital to a physician in the form of medical staff incidental benefits that exceeds the limit set forth in 42 CFR 411.357(m)(5)
9	Remuneration from an entity to a physician (or the immediate family member of a physician) in the form of nonmonetary compensation that exceeds the limit set forth in 42 CFR 411.357(k)(1)
10	Remuneration from an entity to a physician (or the immediate family member of a physician) resulting from a loan to the physician (or the immediate family member of the physician): (1) with an interest rate below fair market value; or (2) on terms that are unavailable from a lender that is not a recipient of the physician’s referrals or business generated by the physician
11	Remuneration from a physician (or the immediate family member of a physician) to an entity resulting from a loan to the entity: (1) with an interest rate below fair market value; or (2) on terms that are unavailable from a lender that is not in a position to generate business for the physician (or the immediate family member of the physician)
12	The referral by a physician owner of a hospital that temporarily expands its facility capacity above the number of operating rooms, procedure rooms, and beds for which the hospital was licensed on March 23, 2010 (or, in the case of a hospital that did not have a provider agreement in effect as of March 23, 2010, but did have a provider agreement in effect on December 31, 2010, the effective date of such provider agreement) without prior application and approval of the expansion of facility capacity as required under Sect. 1877(i)(1)(B) and (i)(3) of the Act and 42 CFR 411.362(b)(2) and (c)
13	Referrals by a physician owner of a hospital that converted from a physician-owned ambulatory surgical center to a hospital on or after March 1, 2020, provided that: (i) the hospital does not satisfy one or more of the requirements of Sect. 1877(i)(1)(A) through (E) of the Act; (ii) the hospital enrolled in Medicare as a hospital during the period of the public health emergency described in section II.A of this blanket waiver document; (iii) the hospital meets the Medicare conditions of participation and other requirements not waived by CMS during the period of the public health emergency described in section II.A of this blanket waiver document; and (iv) the hospital’s Medicare enrollment is not inconsistent with the Emergency Preparedness or Pandemic Plan of the State in which it is located
14	The referral by a physician of a Medicare beneficiary for the provision of designated health services to a home health agency: (1) that does not qualify as a rural provider under 42 CFR 411.356(c)(1); and (2) in which the physician (or an immediate family member of the physician) has an ownership or investment interest
15	The referral by a physician in a group practice for medically necessary designated health services furnished by the group practice in a location that does not qualify as a “same building” or “centralized building” for purposes of 42 CFR 411.355(b)(2)
16	The referral by a physician in a group practice for medically necessary designated health services furnished by the group practice to a patient in his or her private home, an assisted living facility, or independent living facility where the referring physician’s principal medical practice does not consist of treating patients in their private homes
17	The referral by a physician to an entity with which the physician’s immediate family member has a financial relationship if the patient who is referred resides in a rural area
18	Referrals by a physician to an entity with whom the physician (or an immediate family member of the physician) has a compensation arrangement that does not satisfy the writing or signature requirement(s) of an applicable exception but satisfies each other requirement of the applicable exception, unless such requirement is waived under one or more of the blanket waivers set forth above

Source: https://www.cms.gov/files/document/covid-19-blanket-waivers-section-1877g.pdf

Given the COVID-19 pandemic, in general, most healthcare entities were stretched beyond their capacities to provide clinical care. The pandemic entailed an increased influx of patients admitted into healthcare entities, both COVID-19 afflicted and otherwise. There was a predominant national need for providers and the entire healthcare workforce to care for the above influx of patients in tertiary care environments. Healthcare entities were observed to be overstretched because of rapidly consumed hospital resources and medical supplies, owing to the sheer volume of patients admitted at outpacing numbers.

In such circumstances, there was a dire need of flexibility in clinical care. The above situation, additionally, necessitated rendering clinical care in the presence of legalities that were not self-limiting in nature. CMS, contextually, issued 18 blanket waivers to the Stark Law in the presence of the nationwide and public health emergency. CMS, therefore, permitted certain financial arrangements, authorized certain forms of provider remuneration, waived certain sanctions for patient self-referrals, and thereby, protected eligible providers from medicolegal liabilities.

These 18 blanket waivers have trifurcated functions categorized into three segments [13]. These three segments that are solely related to “COVID-19 purposes” strategically ensure the: (1) availability of healthcare items and services, (2) reimbursement for providing such items and services in good faith, and (3) exemption from sanctions arising from self-referral liabilities [13]. These segments are applicable to selective beneficiaries enrolled in Medicare, Medicaid, and CHIP programs [13]. These blanket waivers, furthermore, are applicable to eligible providers that provide healthcare items and services to eligible beneficiaries, exclusively, in the COVID-19 pandemic [13].

In further detail, these blanket waivers ensure that sufficient healthcare items and services are available to meet the needs of program beneficiaries [13]. The above waivers may reimburse eligible providers that furnish healthcare items and services in good faith to program beneficiaries but are unable to comply to one or more regulations of the Stark Law, while doing so, in COVID-19 situations [13]. Blanket waivers, moreover, exempt providers from selective sanctions, and thus, buffer those adhering to the regulations of the Stark Law, fraud, and abuse rules [13].

At first glance, blanket waivers #1 through #11 specify terms and conditions of remuneration and charges [26]. Blanket waivers #12 through #14 specify the same for referrals from physician owners of hospitals and mention those for physicians that have an ownership or investment interest [26]. Blanket waivers #15 and #16 are contextual to patient self-referrals by physicians in group practice settings [26]. Blanket waiver #18, conclusively, waives sanctions arising from the Stark Law in the absence of a written or signed document detailing compensation arrangement [26].

In the forthcoming content, this review paper explores each blanket waiver, one-by-one, to grasp the scope of its functions specific to expanding remuneration for self-referrals, safeguarding physicians’ ownership (or investment interests) and financial arrangements, along with waiving certain sanctions applicable to the above law, and thereby, protecting eligible physicians.

Blanket waiver #1 administers no Stark Law penalty towards remuneration given to the physician (or an immediate family member; hereinafter, a relative) for services he or she personally performs [13, 26, 28]. The above waiver, additionally, permits healthcare entities to pay the physician or relative above or below the Fair Market Value (FMV) for services they personally perform. The above waiver, therefore, protects the entity to additionally compensate physicians employed therein. In the event the entity decides to pay the physician increased salary to bridge provider shortage, arising from overtime or hazard pay, then the above waiver imposes no penalty.

Blanket waivers #2 and #3 authorize the entity paying rental charges below the FMV for occupying and using office space and medical equipment, respectively, to the physician [13, 26, 28]. This payment below the FMV is applicable for leasing office space equipment to provide clinical care. The above waiver helps entities as tenants retain their office space and leverage equipment, in the event they become financially constrained because of the pandemic. These waivers do not protect payments for office space that exceed the FMV. Similarly, they are not applicable to payments for the use of medical equipment that exceeds the FMV.

Blanket waiver #4 permits the entity to remunerate the physician (or relative) an amount below the FMV for healthcare items or services that the entity buys from the latter [13, 26, 28]. The purpose of this waiver is to avoid overpaying for any healthcare item or service during the pandemic. This waiver, moreover, permits restocking healthcare items or services without overpaying for those during the pandemic.

Blanket waivers #5 and #6 authorize the physician (or relative) paying rental charges that are below the FMV for leasing office space and equipment, respectively, from the entity [13, 26, 28]. This waiver does not apply to payments exceeding the FMV. Blanket waivers #2, #3, #5, and #6, therefore, permit entities/physicians, as landlords, to accept rental rates below the FMV for office space and equipment if tenants are financially restrained because of the pandemic. These provisions also render the landlord eligible to provide office space and equipment to the tenant at no extra charge to sustain the surge of patients.

Blanket waiver #7 permits a physician (or relative) paying an amount below the FMV to an entity for using the latter’s premises [13, 26, 28]. The same also permits the former paying an amount below FMV to an entity for buying healthcare items or services from them.

Blanket waivers #8 and #9 permit medical staff and physicians (or relatives) receiving incentives in the form of incidental benefits and non-monetary compensation, respectively, during the pandemic [13, 26, 28]. The above two waivers, therefore, are medical staff benefit and non-monetary physician compensation waivers. The medical staff benefit waiver allows the entity to incentivize medical staff benefits that exceed the $36-per-item limit set forth in 42 CFR § 411.357(m)(5) [13, 26, 28]. The non-monetary physician compensation waiver allows the entity to incentivize physicians’ non-monetary compensation that exceeds the $423 annual limit set forth in 42 CFR § 411.357(k)(1) [13, 26, 28]. The above waiver, additionally, makes provisions to facilitate telehealth, free continuing medical education lectures on COVID-19 training, transportation, childcare, and meals [13, 26, 28].

Blanket waivers #10 and #11 permit remuneration between a physician (or relative) and entity by/to the opposite party to receive monetary loan payments [13, 26, 28]. The above two waivers are referred as monetary loan waivers. The extended loan is permitted to have two provisions [13, 26, 28]. First, the above loan is permitted to have an interest rate less than the FMV [13, 26, 28]. Second, it can be on terms that are unavailable from any other lender or third party with whom the physician (or relative) and entity has associations [13, 26, 28]. The above waivers, therefore, assist with solving liquidity and bankruptcy issues that may arise because of the pandemic.

Blanket waiver #12 permits those referrals from a physician owner that temporarily expands the capacity of the facility [13, 26, 28]. The above waiver is referred as a facility expansion waiver. In this case, this waiver permits expansion of the number of operating rooms, procedure rooms, and beds [13, 26, 28]. The above expansion is applicable to those hospitals licensed on March 23, 2010, or for entities whose provider agreements were in effect on December 31, 2010 [13, 26, 28]. The above waiver does not sanction hospitals for expanding its capacity with no prior application and approval [13, 26, 28].

Blanket waiver #13 permits referrals from physician-owners to hospitals that were previously ambulatory surgery centers (ASCs) [13, 26, 28]. This waiver is referred as a physician-owner referral waiver. The above waiver applies to those hospitals that converted from ASCs on or after March 1, 2020 [13, 26, 28]. There are four conditions to this waiver being applicable [13, 26, 28]. First, the hospital should not satisfy one or more requirements of the Stark Law stated in Sect. 1877(i)(1)(A) through (E) [13, 26, 28]. Second, the hospital should be converted to a Medicare hospital [13, 26, 28]. Third, the hospital should meet Medicare conditions of participation [13, 26, 28]. Fourth, the hospital’s enrollment should be consistent with the Emergency Preparedness or Pandemic Plan of the state in which it is located [13, 26, 28].

Blanket waiver #14 permits a physician to refer Medicare patients for DHS to a home health agency [13, 26, 28]. This waiver is referred to as a home health agency referral waiver. The above waiver permits referrals to home health agencies on the basis that: (1) the home health agency does not qualify as a rural provider stated in 42 CFR 411.356(c)(1), and (2) physicians (or relatives) creating such referrals are either owners or have investment interests in those home health agencies [13, 26, 28].

Blanket waiver #15 permits a physician in a group practice to create referrals for beneficiaries needing medically necessary DHS [13, 26, 28]. This waiver is referred to as the group practice referral waiver. In the above waiver, however, location does not qualify for all requirements set forth in the “same building” or “centralized building” definitions [13, 26, 28].

Blanket waiver #16 permits a physician in a group practice to order services for program beneficiaries at their homes and assisted- or independent-living facilities for medically necessary DHS [13, 26, 28]. This waiver is referred to as the private home, assisted- or independent-living facility waiver [13, 26, 28]. The above waiver is particularly applicable to those beneficiaries who are isolated or observing social distancing in their homes. The eligible group practice, nevertheless, needs to satisfy all the requirements of 42 CFR 411.352 [13, 26, 28].

Blanket waiver #17 permits a physician to refer beneficiaries to an entity in which the physician’s relative has a financial interest [13, 26, 28]. This waiver is referred to as the rural area referral waiver. All the same, it applies when the beneficiary resides in a rural area [13, 26, 28].

Blanket waiver #18 permits the physician to be compensated for referrals to an entity in which the physician (or relative) has a compensation arrangement which may not be specifically printed or signed as a formal contract [13, 26, 28]. This waiver is referred to as the physician compensation waiver in the absence of a written or signed compensation arrangement [13, 26, 28]. This waiver removes the written and signed requirement in a referral arrangement to grant flexibility in compensation [13, 26, 28].

It is important to state some limitations of this study that may prospectively point towards future studies furthering this topic. First, the above paper is limited to curating the legislative and regulatory history of singularly the Stark Law. Second, this study is limited to exploring only the pandemic-related blanket waivers. Observing changes in healthcare practices, at the macro-level, from the evolution of the above law would have added dimension to this study. Third, this study limits itself to providing a bird’s eye overview of the Stark Law from the nascent to its more recent stages. Fourth, if this study had to be explained contextual to the applications of the above law on billing and coding of charges, at the micro-level, then it would probably have expanded the scope of this study. Finally, this paper is limited to curating tabulated updates of the Stark Law until the COVID-19 episode and not its impending ones.

## Conclusion

There are some avenues for future work on this topic aligning with the above limitations. First, there are additional healthcare regulations such as the Anti-Kickback Statute and False Claims Act. A new study, therefore, explaining the evolution of the above two laws would be an avenue for future work. Second, a study explaining the effects of the said law on the overall changes in healthcare practices, at the macro-level, potentially is an insightful one. Third, a more specific study focusing on how this law modulated changes in medical/surgical specialties on patient care as it evolved would also be an informative one. Fourth, a study selectively reviewing how changes in the above law may have impacted clinical billing and coding practices contextually deepens our understanding of this law. Finally, curating the Stark Law’s future initiatives, as they are available, would be keeping in pace with its developments over time.

The purpose of this paper was to review the above law through the lens of its chronological evolution and COVID-19 blanket waivers. In the first spectrum, this review article describes how the above law evolved to its present form in a series of curated developments.

In the second spectrum, this review paper explores details and functions of those developments in forms of amendments and enactments, given the entire gamut of its evolution. In this process, this paper describes above law’s transition from Stark I to Stark II, noting that the latter is more prevalent and currently practiced. Furthermore, it made notes on how CMS insightfully intervened by implementing multiple blanket waivers. CMS, in this process, assuaged prospective convolutions in remuneration, self-referrals, and salvaged sanctions thus arising from the ongoing pandemic.

Finally, this review focuses on how the above blanket waivers were, are, and will continue to be instruments to further buffer our physicians and the healthcare workforce as long as this or any other pandemic continues to be part of our lives.

## Data Availability

Not applicable.
